# The NLRP3 inflammasome in traumatic brain injury: potential as a biomarker and therapeutic target

**DOI:** 10.1186/s12974-020-01778-5

**Published:** 2020-04-06

**Authors:** William T. O’Brien, Louise Pham, Georgia F. Symons, Mastura Monif, Sandy R. Shultz, Stuart J. McDonald

**Affiliations:** 1 Department of Neuroscience, Monash University, Melbourne, VIC Australia; 2 Department of Physiology, Anatomy and Microbiology, La Trobe University, Bundoora, VIC 3086 Australia; 3 Department of Neurology, Alfred Health, Melbourne, VIC 3004 Australia; 4 Department of Neurology, Melbourne Health, Melbourne, VIC 3004 Australia; 5 Department of Physiology, The University of Melbourne, Melbourne, VIC 3052 Australia; 6 Department of Medicine, University of Melbourne, Melbourne, VIC 3052 Australia

**Keywords:** Neuroinflammation, TBI, Concussion, Mild traumatic brain injury, Chronic traumatic encephalopathy, Microglia, IL-1β, Cytokine, Caspase-1, IL-18

## Abstract

There is a great clinical need to identify the underlying mechanisms, as well as related biomarkers, and treatment targets, for traumatic brain injury (TBI). Neuroinflammation is a central pathophysiological feature of TBI. NLRP3 inflammasome activity is a necessary component of the innate immune response to tissue damage, and dysregulated inflammasome activity has been implicated in a number of neurological conditions. This paper introduces the NLRP3 inflammasome and its implication in the pathogenesis of neuroinflammatory-related conditions, with a particular focus on TBI. Although its role in TBI has only recently been identified, findings suggest that priming and activation of the NLRP3 inflammasome are upregulated following TBI. Moreover, recent studies utilizing specific NLRP3 inhibitors have provided further evidence that this inflammasome is a major driver of neuroinflammation and neurobehavioral disturbances following TBI. In addition, there is emerging evidence that circulating inflammasome-associated proteins may have utility as diagnostic biomarkers of neuroinflammatory conditions, including TBI. Finally, novel and promising areas of research will be highlighted, including the potential involvement of the NLRP3 inflammasome in mild TBI, how factors such as biological sex may affect NLRP3 activity in TBI, and the use of emerging biomarker platforms. Taken together, this review highlights the exciting potential of the NLRP3 inflammasome as a target for treatments and biomarkers that may ultimately be used to improve TBI management.

## Background

Traumatic brain injury (TBI) is a subset of acquired brain injury which is induced by an external mechanical force sustained to the head or neck [1]. Although commonly described as a silent epidemic, TBI is reported to be one of the leading causes of long-term disability and equates to a global annual economic burden of an estimated $400 billion [2]. TBI is a highly heterogeneous injury that can cause a range of temporary or permanent neurological alterations [3] and is often categorized into three injury severities: mild, moderate, and severe. These classifications are most commonly defined utilizing the Glasgow coma scale [4]. While TBIs on the mild spectrum were traditionally considered an innocuous injury, recently there has been developing awareness of the potential long-term implications of mild TBI (mTBI), and particularly in relation to repeated mTBIs. The most well publicized long-term implication of repeated mTBIs is the potential development of chronic neuroinflammatory-related conditions such as chronic traumatic encephalopathy (CTE), Alzheimer’s disease, Parkinson’s disease, depression, and anxiety [5–9].

Despite extensive basic and clinical science research on TBI to date, no therapeutic interventions have been successfully implemented to improve patient outcomes [10]. A key factor that has contributed to previous translational failures in TBI is the lack of a detailed understanding of the complex underlying cellular and molecular sequelae. TBI is considered a “biphasic injury” characterized by an initial primary injury and a delayed secondary injury [11]. Primary injury refers to the immediate damage which is caused directly by the mechanical injury, whereas secondary injury refers to further damage due to the pathophysiological changes induced by the primary injury [12]. As secondary injuries can be initiated minutes to hours following injury, and can persist for months to years [13], a greater understanding of the mechanisms of secondary injury may facilitate the discovery of treatments that can improve TBI outcomes. Moreover, a greater understanding of the underlying pathophysiology may facilitate the discovery of diagnostic and prognostic biomarkers of TBI.

Among the different mechanisms postulated to contribute to secondary injury, a neuroinflammatory response characterized by the release of pro-inflammatory mediators and activation of microglia and astrocytes may be universal across TBI subtypes [14–17]. This review will focus on the neuroinflammatory response following TBI, with particular attention to the potentially central role played by a complex of proteins known as the nucleotide-binding oligomerization domain-like receptor pyrin domain-containing-3 (NLRP3) inflammasome.

### Neuroinflammation and TBI

Neuroinflammation is a key cellular and molecular feature of the central nervous system (CNS) response to insults such as a trauma [18]. Microglia, the resident innate immune cells of the CNS, are known to be mediators of the neuroinflammatory response that occurs following TBI [15, 16, 19]. The activation of these cells induces a multitude of inflammatory cascades, including the production and release of downstream pro-inflammatory cytokines such as interleukin (IL)-1β [20]. As such, microglia play a critical role in the CNS immune defense [21]. While neuroinflammation has a crucial neuroprotective role, a dysregulated or persistent neuroinflammatory response may contribute to neurological symptoms and neurodegeneration [22]. For example, it is postulated that dysregulated neuroinflammation likely plays a key role in the aftermath of even mTBIs, and may underlie the persistent post-concussive symptoms that occur in 10–15% of mTBI cases [23]. In addition, with increasing evidence that chronic neuroinflammation can trigger various neuropathological changes including hyperphosphorylation of tau and neuronal apoptosis [24, 25], neuroinflammation may be a key mechanism underlying the increased risk for neurodegenerative diseases for those with a history of TBI [14, 26].

One family of important regulators of the innate immune system is the NOD-like receptors (NLRs) [27]. NLRs are a family of cytosolic pattern recognition receptors typically formed by three components: a sensor molecule, an adaptor protein, and an effector component. Following activation, these subunits combine to form a pro-inflammatory, multiprotein complex termed an inflammasome [28]. Among the multiple NLRs expressed in mammals, the NLRP3 has been the most extensively studied.

### The NLRP3 inflammasome

**Fig. 1:**
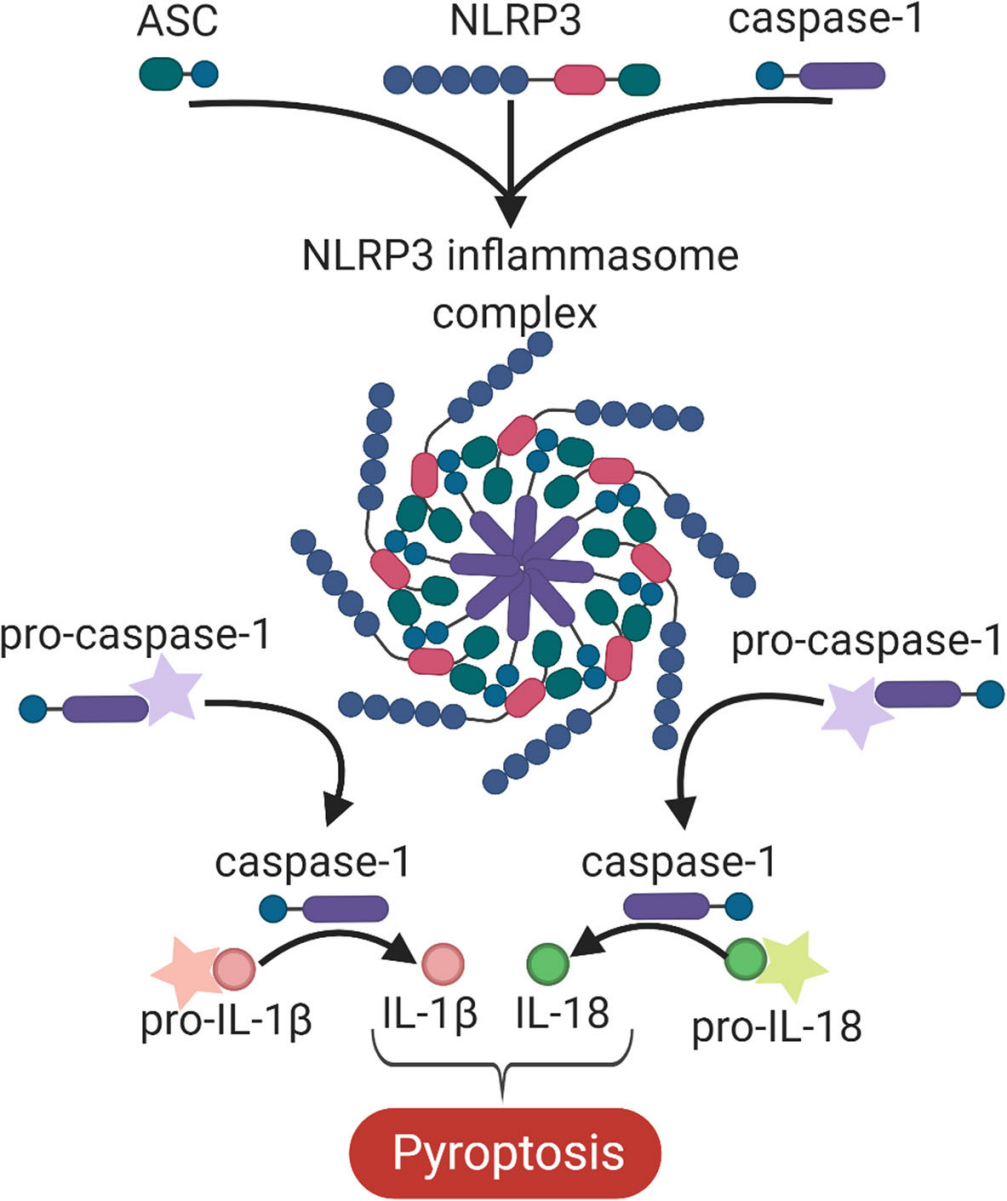
Formation of the NLRP3 inflammasome. Activation of the NLRP3 inflammasome involves the constituent molecules of the NLRP3 inflammasome (i.e. NLRP3, ASC and caspase-1) binding to form a complete NLRP3 inflammasome complex. This inflammasome complex allows the cleavage of pro-caspase-1 into its active isomer, caspase-1, which in turn cleaves pro-IL-1β and pro-IL-18 to their active isomers IL-1β and IL-18 respectively. The increase in these pro-inflammatory proteins ultimately leads to pyroptosis

The NLRP3 inflammasome is a multiprotein complex, composed of three protein subunits: a sensor molecule, NLRP3, an adaptor protein, ASC, and an effector protein, caspase-1 (Fig. 1) [29]. The functional regulation of an active NLRP3 inflammasome is a two-step process; a non-activating “priming” stimulus is firstly required to initiate expression of key inflammasome components, followed by a secondary “activating” stimulus that results in inflammasome oligomerization [30, 31]. Inflammasome priming includes the transcriptional upregulation of NLRP3 and pro-IL-1β, as well as post-translational modifications of NLRP3 that stabilize the inactive protein in a signal-component state. These molecules are inactive until a subsequent (or prolonged) stimulus occurs. The subsequent activation induces the assembly of NLRP3 constituent proteins into the complete NLRP3 inflammasome. This process involves the oligomerization of NLRP3 proteins via homotypic interactions between two NLRP3 proteins, which then recruit and bind ASC. The ASC domain of the partially assembled inflammasome then cleaves pro-caspase-1 into its active isomer, caspase-1, and subsequently binds caspase-1 to form a complete NLRP3 inflammasome. Seven NLRP3 inflammasome molecules are recruited and bind together to form a ring structure. This structure allows the self-cleavage and further activation of pro-caspase-1 proteins into caspase-1. Caspase-1 then facilitates IL-1β and IL-18 maturation via the cleavage of their inactive pro-isomers (pro-IL-1β and pro-IL-18) into their active formation [28, 32]. These cytokines are involved in the innate immune response to infection and trauma, creating a generalized pro-inflammatory environment [33]. As such caspase-1, IL-1β, and IL-18 are commonly utilized in research as indicators of NLRP3 activation. While some NLRP3 inflammasome activity is a necessary component of the innate immune response to pathogens and tissue damage [34], excessive NLRP3 inflammasome activity can lead to a form of cell necrosis known as pyroptosis [35].

**Fig. 2:**
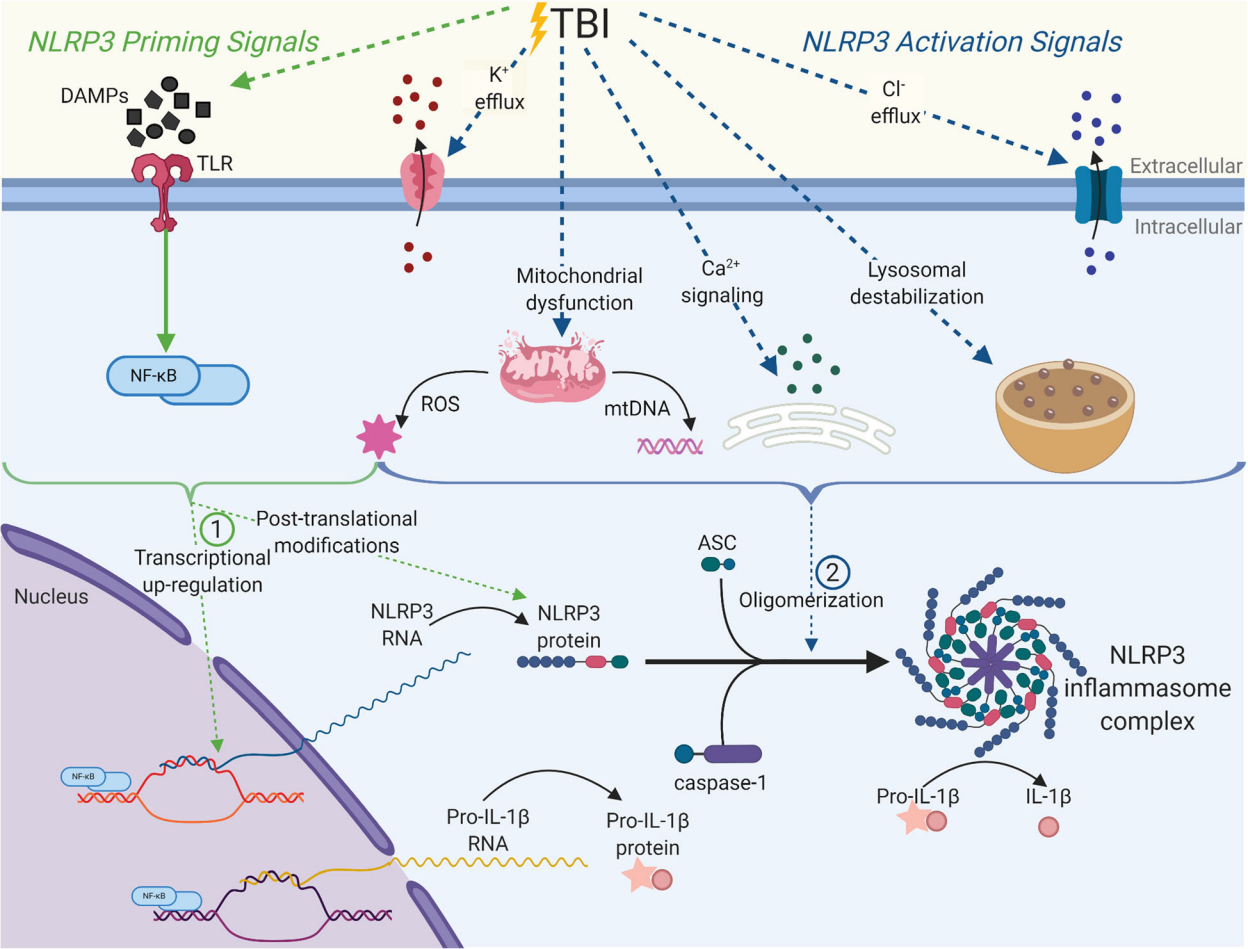
Potential NLRP3 inflammasome priming and activation 1 following TBI. TBI is known to induce an array of molecular changes that may trigger the two-step activation of the NLRP3 inflammasome. (1) Priming of the inflammasome induces transcriptional up-regulation of NLRP3 and pro-IL-1β as well as post-translational modifications of the NLRP3 protein. The most commonly investigated priming signals in the context of sterile trauma is the recognition of DAMPs to induce TLR-NF-κB signalling. DAMPs such as ROS, HMGB1, extracellular matrix molecules and heat shock proteins are known to prime the NLRP3 inflammasome and have also been shown to be up-regulated following TBI. (2) Activation of the inflammasome occurs following priming, and involves the formation of the NLRP3 inflammasome from its constituent proteins (NLRP3, ASC and caspase-1). TBI features a range of endogenous changes that can serve as activating signals, including but not limited to: ionic changes such as potassium and chloride efflux, sodium and calcium efflux, altered calcium signalling, lysosomal destabilisation and products of mitochondrial dysfunction such as mitochondrial DNA and ROS. Importantly, some signals have been shown to upregulate both priming and activation of the NLRP3 inflammasome. This complete inflammasome complex ultimately results in the release of IL-1β.

Multiple signals are known to activate the NLRP3 inflammasome. The signals most commonly investigated are damage-associated molecular pattern (DAMP) and the pathogen-associated molecular pattern molecules [36]. In response to trauma, DAMPs such as reactive oxygen species (ROS) [37], high mobility group box 1 (HMGB1) [38], extracellular matrix molecules [39], and heat shock proteins [40] are known to promote priming of the NLRP3 inflammasome through toll-like receptor (TLR) and NF-κB signaling [30]. In addition, trauma can produce a range of activating signals, including but not limited to the following: ionic changes such as potassium and chloride efflux, sodium and calcium efflux, altered calcium signaling [41–44]; the presence of extracellular ATP [45]; lysosomal destabilization [46]; and products of mitochondrial dysfunction such as mitochondrial DNA and ROS [47, 48]. The precise stimuli that promote the priming and activation steps are not yet fully understood, and there is now evidence that some stimuli, such as ROS, may be involved in both processes [37, 49, 50]. For detailed reviews on the priming and activation of the NLRP3 inflammasome, the reader is referred to articles by Swanson et al. [31], Patel et al. [49], and Herman et al. [51]. Importantly, the aforementioned priming and activating stimuli have been implicated in the aftermath of TBI (see [52–54] reviews), and as such, may play a key role in generating a significant neuroinflammatory response (see Fig. 2).

The potential contribution of the NLRP3 inflammasome to disease pathogenesis was first investigated after a gain-of-function mutation in the NLRP3 coding gene was described as a possible cause of the inflammatory condition cryopyrin-associated periodic syndrome [55]. Since this first report, there has been vast interest in determining the relationship between NLRP3 activation and other inflammatory conditions including type 2 diabetes, atherosclerosis, and steatohepatitis, among others [56–58]. Furthermore, while the expression of NLRP3 in neurons has been disputed, its expression has consistently been found in microglia and astrocytes [59–61]. As such, NLRP3 activity is receiving growing interest as a contributor to various CNS conditions in which glia-mediated neuroinflammation has been associated with disease progression such as Alzheimer’s disease, stroke, Parkinson’s disease, amyotrophic lateral sclerosis, multiple sclerosis, and pneumococcal meningitis [62–68]. Although the NLRP3 inflammasome was not investigated in the context of TBI until 2013, there is now mounting evidence implicating this inflammasome as a critical component in the pathogenesis of the secondary damage that occurs following TBI.

### The NLRP3 inflammasome and TBI

#### Moderate and severe TBI

The first study to investigate the NLRP3 inflammasome in TBI was performed by Liu et al., with the authors finding an upregulation of NLRP3-related genes and proteins in the cortex of rats within the first week following a moderate TBI (modified weight drop model) [61]. Specifically, they reported an elevation of markers of both inflammasome priming (i.e., NLRP3, ASC, and pro-caspase-1 mRNA and protein) and activation (i.e., caspase-1, IL-1β, and IL-18 protein). Most of these inflammasome markers were upregulated at 6 h following injury, and remained elevated until the experimental end point at 7 days post-injury [61]. Since this initial publication, there has been a surge of studies investigating the NLRP3 inflammasome in the context of pre-clinical TBI. These studies have supported the research performed by Liu et al., consistently showing an upregulation of the NLRP3 inflammasome, at both a gene and protein level following a moderate TBI induced via controlled cortical impact (CCI) [69, 70].

There has been limited research investigating the NLRP3 inflammasome in the context of human TBI. One study performed by Lin et al. found an upregulation of NLRP3, caspase-1, IL-1β, and IL-18 protein in the surgically resected cortex of severe-TBI patients in comparison with that of epilepsy patients [71]. Another recent clinical study analyzed cerebrospinal fluid (CSF) from severe-TBI pediatric patients at four time points post-injury, ranging from < 24 h to > 72 h [72], finding that pediatric TBI patients had higher CSF levels of NLRP3 than their age-matched controls (i.e., patients without TBI who underwent a lumbar puncture to rule out CNS infection). Furthermore, a CSF NLRP3 concentration > 6.63 ng/ml at any time point was associated with poorer neurological outcome as determined by the Glasgow Outcome Scale at 6 months post-TBI. This study provided the first evidence of the potential utility of NLRP3 as a fluid-based prognostic biomarker of TBI outcomes. Taken together, these studies have provided clinical evidence of NLRP3 inflammasome activity in moderate and severe TBI.

#### Mild TBI

There is likely to be considerable overlap with certain aspects of mild, moderate and severe TBI pathophysiology. Evidence is mounting suggesting that neuroinflammation plays an influential role in the aftermath of mTBI, with a number of recent pre-clinical studies showing that microglial and astrocytic activation can also be prominent in this form of injury [73–76]. There is limited clinical research on glial activation following mTBI; however, the emergence of positron emission tomography (PET) tracers that bind to the 18 kDa translocator protein (TSPO) that is associated with microglia has enabled in vivo clinical imaging of microglial activation. Eber et al. found that at 1–2 weeks and 3–4 months post-mTBI, patients who had sustained an mTBI had significantly higher TSPO binding when compared to healthy controls [77]. Additionally, a preliminary study of former athletes with an extensive history of mTBI found significantly higher TSPO expression in comparison to healthy age-matched controls [78]. Combined, these preliminary studies suggest that microglial activation may be a prominent feature of single and repeated mTBI.

Despite evidence indicating that neuroinflammation and specifically, glial activation, contribute to the pathogenesis of TBI, to the best of our knowledge, no studies have directly investigated the role of the NLRP3 inflammasome in mTBI. Studies have instead investigated IL-1β, a protein induced by multiple mechanisms, including activation of NLRP3 inflammasome [31, 79]. One study found that rodents administered a single mTBI had an increase in cortical IL-1β protein levels at 6 h post-impact [80]. Interestingly, no difference was found at 3 h following injury indicating a potential delay in the inflammatory response to mTBI [80]. Furthermore, the influence of repeat mTBI on IL-1β levels has also been investigated. Mice given two mTBIs (via modified weight drop) separated by 3 days had an upregulation of IL-1β mRNA in the forebrain 3 days following the final injury [81]. This upregulation was only temporary, with mRNA levels not significantly different to sham-injured controls when the animals were allowed to recover for 20 days [81]. Similarly, mice given two mTBIs separated by 24 h also had elevated cortical IL-1β protein levels that peaked at 48 h following the final injury and were no different to shams at 7 and 14 days [82].

There is now evidence that neuroinflammation, and specifically glial activation, and IL-1β are prominent in the aftermath of both single and repeated mTBI [78, 80–82]. Nonetheless, while the NLRP3 inflammasome causes the cleavage of IL-1β from its inactive isomer, IL-1β is not specific to the NLRP3 inflammasome. As such, increases in IL-1β may not necessarily indicate NLRP3 inflammasome activity, with future studies required to determine functionality of this inflammasome following mTBI.

### NLRP3 as a therapeutic target for TBI

The aforementioned evidence that TBI can activate the NLRP3 inflammasome has led to the hypothesis that therapies targeting this pathway may be effective for mitigating neuroinflammation and improving TBI recovery. In animal models of other neuroinflammatory-related conditions such as Alzheimer’s disease and stroke, knock-down or knock-out of NLRP3 has been shown to decrease neuroinflammation as well as improve functional outcomes [62, 63]. In addition, a number of recent studies have found that pharmacological treatments that directly or indirectly target this inflammasome can reduce its activity following moderate-to-severe TBI. These treatments are broadly broken into four groups: (i) treatments derived from naturally occurring compounds (e.g., mangiferin [83], omega-3 fatty acids [71], and apocynin [84]); (ii) repurposed medications (e.g., propofol [85], and telmisartan [86]); (iii) inhibitors of NLRP3-associated molecules (e.g., ASC antibodies [87], NF-κB inhibitor, Bay 11-7082 [88–91]); and (iv) specific NLRP3 inflammasome inhibitors (e.g., MCC950 [69], JC-124 [92]). While the first three treatment categories have been shown to decrease NLRP3 inflammasome activity as well as demonstrating a neuroprotective effect, they do not target NLRP3 activation specifically. As such, it is difficult to determine whether the improved outcomes are NLRP3 inflammasome dependent, or whether the NLRP3 inflammasome changes are the result of an alternative mechanism of action (e.g., reduction in activating stimuli). Hence, the NLRP3 specific inhibitors, MCC950, and JC-124, may hold the most promise for unearthing the precise roles of the NLRP3 inflammasome in TBI, and consequently reveal potential therapies aimed to improve TBI outcomes. A table summarizing currently available therapies that have been tested in the context of TBI and shown to directly or indirectly inhibit the NLRP3 inflammasome is displayed below (Table 1).

**Table 1:** Specific and non-specific NLRP3 inhibitors investigated in the context of TBI

Inhibitor	NLRP3 specific?	Pre-clinical TBI studies	Species	Model of TBI
MCC950	Yes (NLRP3 NACHT domain)	[70] [69] [93]	Mouse	CCI
JC124	Yes (unknown)	[92]	Rat	CCI
Bay 11-7082	No	[88–91]	Rat and mouse	CCI, weight-drop, and fluid percussion
Mangiferin	No	[83]	Rat	Blast
Omega-3 fatty acids	No	[71]	Rat	CCI
Apocynin	No	[84]	Mouse	CCI
Propofol	No	[85]	Rat	Blast
Telmisartan	No	[86]	Mouse	Cryogenic

MCC950 is a highly selective and potent NLRP3 inhibitor originally derived from the anti-diabetic drug class, sulfonylurea. MCC950 acts by binding directly to the NACT domain of the NLRP3 protein [94]. Pre-clinical investigations on this compound have determined it to have a suitable bioavailability and pharmacokinetic profile, with good CNS penetration as well as no off-target binding of NLRC4 or the NLRP1 inflammasome [94, 95]. MCC950’s specificity for the NLRP3 inflammasome and high CNS penetrability decreases the likelihood of off-target effects (a common side effect of anti-inflammatory molecules). Systemic administration of MCC950 has demonstrated promising results in pre-clinical studies of ischemic stroke, cerebral hemorrhage, and Alzheimer’s disease [62, 96, 97], with two recent studies also showing some promise in the context of TBI of moderate severity [69, 70]. The first of these studies found that acute treatment with MCC950 (50 mg/kg; intraperitoneal (IP) injection) prevented increases in NLRP3 constituent proteins (NLRP3, caspase-1, ASC, and IL-1β) in the cortex of mice at 24 h following CCI. In addition, caspase-1 and IL-1β levels were decreased in MCC950-treated mice at 72 h post-TBI [69]. These reductions in inflammasome activity were also accompanied by evidence of functional improvement, with treated mice displaying reduced modified neurological severity score (mNSS) compared to vehicle mice at 72 h post-injury [69]. The second study, performed by Xu et al., treated mice with MCC950 via IP injection (10 mg/kg) daily for the first 3 days post-CCI, and every second day thereafter [70]. At 72 h post-CCI, MCC950-treated mice had reduced protein expression of NLRP3, ASC, and caspase-1. Additionally, treated mice had improvements in the mNSS and motor function at three-, seven-, and 14-day post-injury, as well as cognition at 17- and 18-day post-injury [70]. Taken together, these findings suggest efficacy of MCC950 in the acute stages following focal TBI in mice; however, it is unknown whether this compound exerts beneficial effects in other species and TBI models (e.g., diffuse TBI). While systemic MCC950 treatment has shown to be well-tolerated in hypertensive mice for as long as 28 days [98], pre-clinical TBI studies have yet to treat with MCC950 for longer than 7 days. Furthermore, the effects of early MCC950 intervention on chronic TBI recovery have not yet been investigated.

Another NLRP3 inflammasome inhibitor, JC-124, has recently been investigated in the context of TBI [92]. JC-124 was designed though the structural optimization of the anti-diabetic compound glyburide, in order to increase the selectivity for the NLRP3 inflammasome and hence decrease the off-target binding [99]. JC-124 acts by inhibiting the formation of the NLRP3 inflammasome [99]; however, the specific mechanisms through which this occurs are unknown. JC-124 has previously been shown in rodents to reduce inflammation and infarct size following myocardial injury [100]. In the only study on JC-124 and TBI to date, Kuwar et al. found that rats given a moderate CCI followed by acute treatment with JC-124 (100 mg/kg, IP) had reduced expression of NLRP3 inflammasome activation markers at 48 h when compared to vehicle-treated rats. Interestingly, IL-18 levels were not altered by TBI with or without JC-124 treatment [92]. Other TBI studies have also found differential expression patterns of IL-1β and IL-18, with IL-1β being the “initial responder” to injury followed by a “delayed” IL-18 response [61, 101, 102]. As such, inflammasome activation may not result in simultaneous upregulation of downstream cytokines. While no behavioral outcomes were measured in this study, JC-124 treatment was found to reduce lesion volume and the number of degenerating neurons as quantified by Fluoro-Jade B staining.

Although further studies are required, taken together these pharmacological studies have further established the link between the NLRP3 inflammasome and TBI, and suggest that treatments target this pathway may have potential for improving TBI outcomes.

### NLRP3 as a biomarker for TBI

There is a growing interest to discover objective biomarkers that can assist the clinical management of TBI. In particular, fluid-based biomarkers have received much attention for their potential clinical applications, including assisting in TBI diagnosis, determination of injury severity, prediction of outcomes, monitoring of recovery, identification of underlying pathophysiology, and treatment efficacy. To date, most investigations of fluid biomarkers of TBI have focused on proteins released into CSF and blood due to axonal and glial damage; however, given the prominent role of neuroinflammation in TBI, a number of neuroinflammation-associated molecules have also recently emerged as biomarker candidates. In particular, given that astrocyte and microglia reactivity can be prominent following TBI [14–17], and these cell types are the primary cells that express NLRP3 [59, 70], it is hypothesized that NLRP3-associated molecules may have utility as biomarkers of TBI pathophysiology.

While few studies have investigated serum levels of NLRP3 inflammasome-related proteins in the context of neuroinflammatory conditions, protein levels of ASC, caspase-1, IL-1β, and IL-18 were all found to be significantly upregulated in the serum of ischemic stroke patients compared to age-matched healthy controls [103]. Furthermore, levels of caspase-1, IL-1β, and IL-18 were able to delineate between stroke and healthy controls, albeit with a modest degree of sensitivity and specificity [103]. Serum levels of ASC, however, were found to have significant diagnostic potential, demonstrating 100% sensitivity and 96% specificity to detect the presence of cerebral ischemia. Similarly in the context of MS, protein levels of ASC, caspase-1 and IL-18 (but not IL-1β) were upregulated in the serum of multiple sclerosis patients compared to healthy age-matched controls [67], with ASC found to have the greatest diagnostic sensitivity and specificity. Furthermore, circulating ASC levels were found to have moderate ability to predict the severity of multiple sclerosis. In the context of TBI, this same group found that both ASC and caspase-1 (but not IL-1β or IL-18) were significantly upregulated in the serum of severe-TBI patients within the first 48 h of injury, with both proteins having excellent utility for distinguishing between control and TBI patients [104]. Interestingly, CSF levels of IL-18 and ASC were also assessed, with both proteins found to be at upregulated and accurate indicators of TBI. However, as participants enrolled in the CSF arm of the study were not the same as the serum arm, it is impossible to determine a correlation between these two biofluids. A separate study performed by Ciaramella et al. investigated the utility of serum IL-18 as a biomarker of severe-TBI in the chronic stages of recovery [105]. TBI patients at 87.8 ± 12.7 days post-injury had elevated serum IL-18 protein levels compared to age-matched healthy controls. Importantly, the serum IL-18 levels of the TBI patients correlated to the level of cognitive impairment and disability severity as determined by Levels of Cognitive Functioning and the Disability Rating Scale respectively [105].

The aforementioned severe-TBI studies provide the first evidence indicating a potential role for NLRP3-related proteins, particularly ASC and IL-18, as fluid biomarkers of TBI. However, these two proteins are not specific to the inflammasome and as such, without direct assessments of the NLRP3 protein, do not necessarily provide an insight into the role of the NLRP3 inflammasome itself in the aftermath of TBI.

### Future directions

As described above, since 2013, there have been several lines of evidence indicating that the NLRP3 inflammasome is upregulated and contributes to the pathology of TBI. While these reports are promising, there remains multiple gaps in the current literature. These gaps are described below.

mTBI*:* To date, there have been no specific investigations into the NLRP3 inflammasome and its potential contribution to the neuropathological and neurobehavioral effects of mTBIs. In particular, the utility of NLRP3-associated proteins as objective biomarkers of mTBI remains unexplored, but important to investigate given that the diagnosis and management of this form of injury remain notoriously difficult [106, 107]. Furthermore, in the context of sports-related mTBI, collision sports athletes are at risk of experiencing multiple mTBIs across their career. Multiple mTBIs, or repeated mTBIs, have been linked to the development of chronic deficits, including neurodegenerative diseases associated with neuroinflammation, such as CTE [5]. It is not yet known whether the NLRP3 inflammasome is involved in the potential cumulative effects of repeated mTBI; however, as the inflammasome requires a two-step activation (i.e., priming and activation), previous mTBIs may prime the inflammasome, with increased basal NLRP3 expression creating vulnerability for a subsequent mTBI to induce inflammasome activation, and as a result, an exaggerated and prolonged neuroinflammatory response.

#### Temporal changes of the NLRP3 inflammasome

There are inconsistencies in the current literature on the temporal profile of NLRP3 activity following TBI. Greater temporal characterization is required to both understand the contribution of the inflammasome to TBI-related neuropathological and neurobehavioral changes and to identify appropriate windows for assessment of biomarkers and application of treatments. Additionally, while one study has shown behavioral improvements at 21 days post-TBI with NLRP3 inflammasome inhibition [70], currently no investigations have analyzed the NLRP3 inflammasome and its inhibition beyond 7 days post-TBI. As such, future studies need to extend past these acute and sub-acute time points to investigate the role of the NLRP3 inflammasome in the chronic stages of TBI.

#### Effect of NLRP3 inflammasome on TBI pathophysiology

Given the increasing awareness that neuroinflammation can interact with other aspects of TBI pathophysiology, it is likely that alterations or manipulation of the NLRP3 inflammasome will have multiple pathophysiological consequences. For example, recent studies have found that a relationship exists between neuroinflammation, oxidative stress, and blood-brain barrier permeability after TBI [84, 108, 109]. The involvement of the NLRP3 inflammasome in these interactions is not yet known; however, MCC950 treatment TBI was found to reduce the extent of blood-brain barrier damage and apoptosis in the acute stages after in TBI mice [70]. NLRP3 may also interact with tau pathology, a prominent feature of chronic TBI, with Ising and colleagues recently reporting strong evidence of a bi-directional relationship between NLRP3 activation and hyperphosphorylation and aggregation of tau [110].

#### Biological sex and the NLRP3 inflammasome

To date, all animal studies investigating the relationship between the NLRP3 inflammasome and TBI have exclusively utilized male rodents. It is becoming increasingly appreciated that males and females can have different biological and behavioral responses to TBI [111]. Of particular relevance, there is some evidence that the nature of neuroinflammatory responses after TBI may differ between sexes. For example, Villapol and colleagues recently found that the microglial response to moderate-to-severe CCI differed between sexes, with male mice displaying an earlier and more intense microglial activation when compared to female mice [112]. Interestingly, a recent study found that the NLRP3 inflammasome had a sex-dependent effect on post-operative pain, with male but not female NLRP3 knockout mice demonstrating less mechanical hypersensitivity when compared to wild type mice [113]. Although preliminary, these findings suggest that NLRP3-driven pathology may be more prominent in males. On a related note, Thakkar and colleagues recently found that activation of the NLRP3 inflammasome following ischemic brain injury was significantly impaired by estradiol signaling [114]. As such, future studies are warranted to decipher whether the nature and significance of NLRP3 activation following TBI does indeed differ between sexes.

#### Age and the NLRP3 inflammasome

Aging populations represent a significant proportion of all TBI patients, with 2013 reports indicating that adults over the age of 75 accounted for approximately one-third of all TBI-related deaths and hospitalizations [115]. Aging has been strongly associated with an increase in basal inflammation and dysregulation of the innate immune system [116]. Despite this, aged rodents are rarely included in pre-clinical studies [117]. Although the NLRP3 inflammasome is a key driver of the innate immune response [118], to date, no studies have investigated the NLRP3 inflammasome in aged TBI subjects. Future studies directly investigating the contribution of the NLRP3 inflammasome to the pathophysiology of TBI in aging populations is required.

#### Novel biomarkers of the NLRP3 inflammasome

While preliminary evidence implicating inflammasome-associated proteins as biomarkers of TBI is promising, recent technological advances and the emergence of alternative biomarker candidates have created opportunities for discovery of other NLRP3-associated biomarkers. For example, the pro-inflammatory cytokine IL-1β has previously been investigated as a serum biomarker of TBI. This analysis, however, has thus far failed due to the low serum detectability of IL-1β. Recent developments of highly sensitive assays, such as the single molecule array (SIMOA®), have resulted in lower detection limits and the ability to accurately quantify IL-1β in the periphery [119, 120]. Similarly, the detection of NLRP3-related molecules such as ASC has been made possible due to assays available on the Ella Simple Plex System (ProteinSimple) [103, 104]. Nonetheless, investigations into the ultimate utility of these novel biomarkers are somewhat hampered by the low accessibility to these specific immunoassay platforms. However, these assays may enable future investigations of peripheral NLRP3 inflammasome proteins in the context of neuroinflammatory-related conditions. Furthermore, short non-coding strands of RNA termed microRNAs (miRNA) are receiving growing evidence as potential biomarkers of various CNS disorders including Alzheimer’s disease, Parkinson’s disease, and TBI [121, 122]. miRNAs such as miR-223-3p are known regulators of the NLRP3 inflammasome and act at a priming and activation level of NLRP3 formation [123]. miRNAs, which regulate the NLRP3 inflammasome have never previously been investigated in the context TBI. Additionally, the NLRP3 inflammasome and its related proteins are not CNS specific, as the NLRP3 protein has been shown to be elevated in systemic inflammatory disorders including type 2 diabetes, atherosclerosis, and steatohepatitis [56–58]. As such, the implementation of novel techniques including the isolation of molecules contained in CNS-derived extracellular vesicles holds the potential to determine the cellular origin of the detected molecules and hence ensure their relevance to CNS pathology [124].

## Conclusions

TBI is a global health concern; however, there are no proven therapeutic interventions to improve clinical outcomes. Recent findings from human and rodent studies have indicated an upregulation in NLRP3-related molecules following TBI. Moreover, rodent intervention studies have found that specifically inhibiting the NLRP3 inflammasome can mitigate neuroinflammation and improve outcomes following TBI. Additionally, emerging reports suggest that circulating NLRP3 and its associated molecules may function as biomarkers of neuroinflammatory conditions. Although promising, there remains a number of important knowledge gaps, including potential effects of NLRP3 inhibitors such as MCC950 on peripheral immune function and any implications this may have on host-defense mechanisms, as well as the optimal timing and dose of administration after TBI. It is recommended that further research also investigates mTBI, includes variables such as age and biological sex, determines the diagnostic and prognostic ability of inflammasome-associated biomarkers, and further establish if NLRP3-targeted treatments can improve TBI outcomes.

## Data Availability

Not applicable

## Abbreviations

TBI: Traumatic brain injury
mTBI: Mild TBI
CTE: Chronic traumatic encephalopathy
CNS: Central nervous system
NLRP3: Nucleotide-binding oligomerization domain-like receptor pyrin domain-containing-3
IL: Interleukin
NLRs: NOD-like receptors
DAMP: Damage-associated molecular pattern
ROS: Reactive oxygen species
HMGB1: High mobility group box 1
TLR: Toll-like receptor
CCI: Controlled cortical impact
CSF: Cerebrospinal fluid
PET: Positron emission tomography
TSPO: Translocator protein
IP: Intraperitoneal
mNSS: Modified Neurological Severity Score
SIMOA: Single molecule array
miRNA: MicroRNA
